# Exergaming as a promising approach to promote physical activity in children and adolescents during hemodialysis?

**DOI:** 10.1007/s00467-026-07158-0

**Published:** 2026-01-15

**Authors:** Thomas Schmidt, Lene Stegelmann, Marleen Kerstin, Christina Taylan, Bernd Hoppe, Klara Brixius

**Affiliations:** 1 Department for Preventive and Rehabilitative Sports and Performance Medicine, Institute of Cardiology and Sports Medicine, German Sport University Cologne, Am Sportpark Müngerdorf 6, Cologne, 50933 Germany; 2 Schüchtermann-Klinik Bad Rothenfelde, Bad Rothenfelde, 49214 Germany; 3 Pediatric Nephrology, University Hospital of Cologne, Cologne, 50937 Germany; 4 Bonn Center of Pediatric Nephrology, Bonn, 53127 Germany; 5 Department for Molecular and Cellular Sports Medicine, Institute of Cardiology and Sports Medicine, German Sport University Cologne, Cologne, 50933 Germany

**Keywords:** Chronic kidney disease, Hemodialysis, Exergaming, Intradialytic exercise, Exercise therapy, Children

## Introduction

Children and adolescents on hemodialysis typically engage in substantially less physical activity than their healthy peers, clearly falling short of current recommendations [1, 2]. Exergaming, a combination of exercise and gaming, may help transform sedentary dialysis time into active time, thereby increasing motivation and engagement while promoting regular physical activity in this vulnerable population [1, 3, 4].

## Methods

### Exergaming intervention

**Fig. 1:**
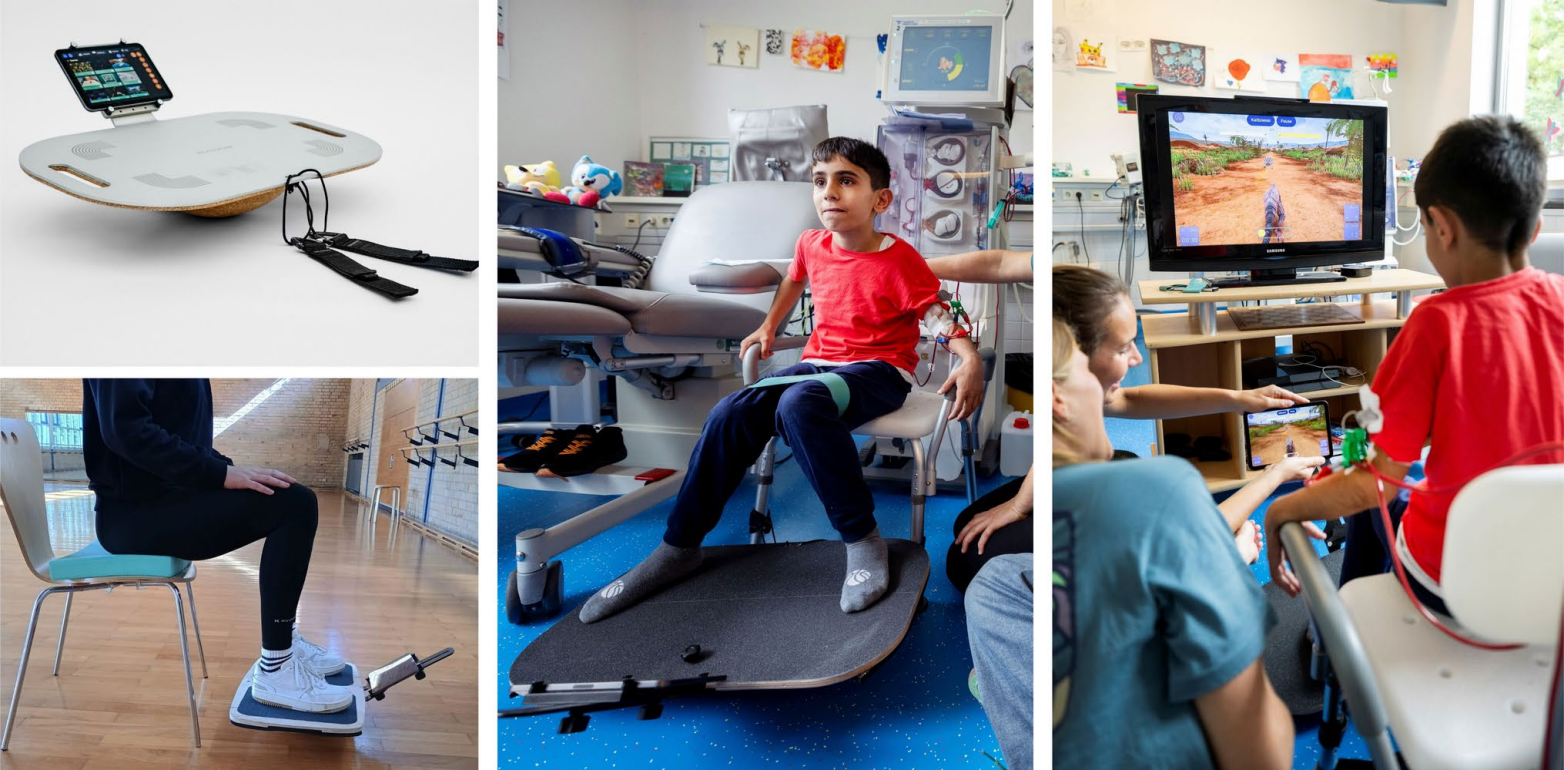
Exergaming during hemodialysis. Children and adolescents control a video game through their movements while seated next to the dialysis beds. Additional elements, such as balance pads or resistance bands, can be used to increase exercise intensity (permissions obtained: top left, ICAROS GmbH; bottom left, own illustration; center and right, photographs by Michael Bause)

Participants used a novel exergaming system that was developed iteratively in cooperation with ICAROS (ICAROS GmbH, Martinsfeld, Germany). In a seated position, they controlled a wobble board–like training device (Icaros Seated Trainer (I.S.T.)) with their legs. A mobile device was mounted at the front of the board; its integrated motion sensors (accelerometer, gyroscope) captured the movement in real time and translated it into actions within a video game. In addition, a monitor screen was positioned in front of the child, on which the video game was displayed live (Fig. 1).

In total, 23 different games were available via the ICAROS app. Depending on the game type (e.g., boxing, skiing, dinosaur hunting), they imposed varying physical and cognitive demands, targeting strength, balance, cognition, reaction, coordination, and endurance. Furthermore, additional elements such as resistance bands and balance pads and cushions were integrated to increase the intensity of muscle activation.

### Prospective exploratory pilot study

This pilot study was conducted at the Department of Pediatric Nephrology, University Hospital Cologne, and at the Bonn Center of Pediatric Nephrology. In addition to standard medical care, pediatric patients participated in an 8-week exergaming intervention (two sessions per week, 20 min each), performed during the first third of the dialysis session. The primary aim was to gain initial experiences and assess feasibility, safety, and acceptability.

## Results and future implications

Nine children and adolescents (22% female; 11.6 ± 3.8 years; 21 ± 31 months on hemodialysis) completed a total of 123 training sessions. No falls, arteriovenous (AV) shunt needle dislodgements, or other serious adverse events were observed during the exergaming sessions. Minor events included pressure alarms (*n* = 22), headache/abdominal pain (*n* = 4), or dizziness/nausea (*n* = 3). In the latter cases, symptoms were already present before training and led to premature termination of the session. All complaints resolved without further consequences. Children, caregivers, and physicians provided consistently positive feedback, and there was a clear wish to continue the exergaming program beyond the pilot study.

### Challenges

- Integration into the dialysis schedule requires workflow coordination.
- Initially, some anxiety and concern were reported, but these gradually subsided.
- Ensuring sufficient space between beds.

### Recommendations and outlook

- Use of a height-adjustable chair with adapted armrests to prevent kinking and dislocation of dialysis lines.
- Further hardware and software optimization possible: refined version of the I.S.T., target-group–specific games, multiplayer mode, VR headset, gradual transition to exercises in a standing position.

Our first experiences suggest that exergaming is a promising and feasible exercise intervention during pediatric hemodialysis and warrants further development and evaluation in larger studies.

## Data Availability

The datasets generated and/or analyzed for this manuscript are available from the corresponding author on reasonable request.
